# Comparative Metabolomics Reveals the Nutritional Merit and Metabolic Basis of a Naturally Occurring White Variant in *Flammulina filiformis*

**DOI:** 10.3390/foods15081373

**Published:** 2026-04-15

**Authors:** Shuangtao Zhang, Baoyu Cui, Shumei Cui, Shiyun Wei, Shunfen Wang, Kunzhi Jia, Chongrong Ke

**Affiliations:** 1 College of Life Sciences, Fujian Agriculture and Forestry University, Fuzhou 350002, China; zhangst2024@163.com (S.Z.);; 2 Jiashu Biotechnology Co., Ltd., Fuzhou 350001, China; 3 Qiyuan Intelligent Manufacturing Biotechnology Co., Ltd., Fuzhou 350001, China; 4 College of Life Sciences, Fujian Normal University, Fuzhou 350117, China; 5 College of Horticulture, Fujian Agriculture and Forestry University, Fuzhou 350002, China

**Keywords:** *Flammulina filiformis*, color variation, natural white strain, quality evaluation, metabolomics

## Abstract

The color of *Flammulina filiformis* is an important commercial trait, and most natural varieties are yellow. This study focused on a natural white variant strain (CN-01) and a yellow strain (JSH17) of *F. filiformis*. We conducted physiological index measurement and untargeted metabolomics analysis to systematically evaluate its nutritional quality and preliminarily investigate the metabolic differences associated with its white phenotype. The results showed that the total free amino acid content and sweetness intensity of strain CN-01 were superior to those of strain JSH17, although its bioactive components were comparatively lower. Metabolomics analysis revealed that the differential metabolites between the two strains were predominantly enriched in pathways related to amino acid metabolism, energy metabolism, fatty acid metabolism, and glutathione metabolism. Notably, the aromatic amino acid biosynthesis pathway, which is closely associated with pigment synthesis, was not significantly activated in the white strain, likely serving as the key metabolic reason for the formation of its white phenotype. This study provides a scientific basis for resource evaluation and utilization of natural white *F. filiformis* and elucidates the biochemical mechanisms underlying its color variation from a metabolic perspective.

## 1. Introduction

*Flammulina filiformis* is a widely popular edible and medicinal fungus. Its fruiting bodies are nutritionally rich, with high protein content, essential amino acids, and various bioactive substances, exhibiting specific functions in anti-tumor, antihypertensive, cholesterol-lowering, antioxidant, and fatigue-relieving activities [1]. In 2023, the total production of *F. filiformis* in China reached 1.8738 million tons, ranking it among the leading cultivated edible fungi varieties [2]. With the diversification of market demand and the increasing emphasis on quality, the selective breeding and quality improvement of *F. filiformis* have become research hotspots [3].

Currently, the mainstream commercial strains are yellow or light yellow varieties, while the common white *F. filiformis* is mostly an artificially bred “albino” variety [4]. Notably, rare wild white variant strains also exist in nature. The differentiation of color is essentially an intuitive manifestation of the systemic remodeling of metabolic networks within an organism [5] and is often closely associated with alterations in biosynthetic pathways of pigments such as carotenoids [6] and flavonoids [7]. These secondary metabolic pathways, in turn, have extensive interactions with primary metabolism, including amino acid and carbohydrate metabolism [8]. Therefore, systematically comparing the metabolic similarities and differences between natural white and yellow strains is crucial for scientifically evaluating their quality disparities and elucidating the mechanism underlying the formation of the white phenotype.

Metabolomics is an emerging omics technology developed after genomics, transcriptomics, and proteomics. It primarily focuses on the global and dynamic changes of endogenous small-molecule metabolites within an organism, thereby directly reflecting the ultimate biochemical phenotype [9]. Compared with genomics and transcriptomics, metabolomics enables rapid screening of differential metabolites across samples, providing a direct and real-time reflection of the physiological state of an organism at a specific time and under specific environmental conditions [10]. Currently, metabolomics technology has been widely applied in trait analysis of various organisms, including *Blakeslea trispora* [11], *Cordyceps militaris* [12], and *Stropharia rugosoannulata* [13]. However, systematic research on the nutritional quality of natural white *F. filiformis* and the metabolic basis underlying its white coloration remains lacking.

Therefore, this study focused on a natural white variant strain (CN-01) and a yellow strain (JSH17) of *F. filiformis*. Through gene sequence comparison, morphological observation, physiological index measurement, and untargeted metabolomics analysis, we scientifically evaluated the nutritional and flavor value of the natural white *F. filiformis* relative to the typical yellow strain and preliminarily investigated the potential biochemical mechanism underlying its white phenotype formation. This research not only provides guidance for the domestication and utilization of rare wild strains but also offers a new theoretical basis and material foundation for the innovation and improvement of *F. filiformis* germplasm resources in China.

## 2. Materials and Methods

### 2.1. Materials and Methods

The *Flammulina filiformis* strains used in this study were provided by Fuzhou Jiashu Biotechnology Co., Ltd. (Fuzhou, China). The yellow strain JSH17 was collected from a natural environment in Hangzhou (China, longitude 120.144061, latitude 30.289935) and obtained through tissue isolation. The white variant strain CN-01 was collected using the same method in Nanping (China, longitude 118.0777547, latitude 27.3864812). The cultivation substrate required for *F. filiformis* culture and other reagents used in the experiments were of domestic analytical grade.

### 2.2. Cultivation Substrate and Parameters for Flammulina filiformis

The *F. filiformis* strains JSH17 and CN-01 were cultivated following an industrial cultivation model. The substrate (65% sawdust, 15% cottonseed hull, 15% wheat bran, 2% lime, 1.5% gypsum powder, 1.5% superphosphate; 60–65% water content) was mixed, filled into 250 mL plastic bottles (200 g per bottle), sterilized at 121 °C for 3 h, and inoculated with liquid spawn under aseptic conditions.

During mycelial growth, cultures were maintained at 16–18 °C with 75–80% humidity in darkness until full colonization, followed by a one-week maturation period. The surface was then scratched to promote fruiting body initiation. For primordium induction, temperature was gradually reduced from 14 to 16 °C to 12–14 °C, humidity was maintained at 85–98%, and light intensity was set to 300 lx. After primordia formation, humidity was lowered to 85–90%. During fruiting body development, temperature was further reduced to 6–8 °C, humidity was controlled at 75–93%, and light cycles (50 lx, 5 min light/2 h dark) were applied.

### 2.3. Identification of Flammulina filiformis Strains

Genomic DNA was extracted from fruiting bodies using a fungal genomic DNA isolation kit (Sangon Biotech, Shanghai, China). The internal transcribed spacer (ITS) region was amplified by PCR using the universal primers ITS4 (5′-TCCTCCGCTTATTGATATGC-3′) and ITS5 (5′-GGAAGTAAAAGTCGTAACAAGG-3′). The amplified products were purified and sequenced by Sangon Biotech Co., Ltd. (Shanghai, China). The resulting sequences were subjected to BLAST alignment against the NCBI database (https://www.ncbi.nlm.nih.gov/). Relevant sequences of *Flammulina* taxa were downloaded from GenBank and aligned with the sequences of strains JSH17 and CN-01 using MUSCLE within PhyloSuite. A Bayesian phylogenetic tree was constructed using MrBayes and visualized using the iTOL website (https://itol.embl.de/), with *Rhodotus asperior* selected as the outgroup.

### 2.4. Observation of Fruiting Body Microstructure

Tissue sections were prepared from the pileus and stipe of *F. filiformis* fruiting bodies of strains JSH17 and CN-01. The sections were placed in a 2.5% glutaraldehyde solution and fixed overnight at 4 °C. After multiple rinses with 0.1 mol/L phosphate-buffered saline (PBS, pH 6.8), the samples were dehydrated using a graded ethanol series and then transferred to isoamyl acetate for an additional 15 min of dehydration. The dehydrated samples were subjected to vacuum freeze-drying (FD-1A-50, BIOCOOL, Beijing, China). Subsequently, their ultrastructural features were observed and analyzed using a scanning electron microscope (SEM, ZEISS ULTRA 55, Oberkochen, Germany).

### 2.5. Determination of Physiological Indicators

#### 2.5.1. Electronic Tongue Analysis

A 5.0 g sample of fruiting bodies was thoroughly ground in a mortar. Subsequently, distilled water was added (1:5, *w*/*v*) and mixed uniformly. The mixture was heated in a constant temperature water bath at 100 °C for 5 min. After cooling, it was centrifuged at 8000 rpm for 15 min at 4 °C. The supernatant was collected and stored at 4 °C for further analysis. The supernatant was collected for analysis using a SA402B electronic tongue (Insent, Hiratsuka, Japan) with appropriate reference and cleaning solutions. Taste measurement time was 30 s per sample, with sensor cleaning for 3 s between measurements. Each sample was analyzed 3–5 times, with the last three measurements used for data analysis.

#### 2.5.2. Soluble Sugar and Protein Content

Samples (0.1 g) were homogenized with 1 mL distilled water. For soluble sugar extraction, the homogenate was boiled for 10 min; for soluble protein extraction, the homogenate was ground on ice with pre-cooled distilled water. All homogenates were centrifuged at 8000–10,000 rpm for 10 min at 4 °C. The supernatants were appropriately diluted with distilled water. Soluble sugar and protein contents were determined using the Plant Soluble Sugar Content Assay Kit and the BCA Protein Content Assay Kit (Sinobestbio, Shanghai, China), respectively.

#### 2.5.3. Total Phenolic and Flavonoid Content

Dried and powdered samples (0.1 g, 40-mesh) were extracted with 1 mL extraction solution by ultrasonication (300 W, 5 s on/8 s off, 60 °C, 30 min). After centrifugation at 12,000 rpm for 10 min at 4 °C, the supernatant was adjusted to 1 mL with extraction solution. Total phenolic and flavonoid contents were determined using Plant Total Phenol and Total Flavonoid Content Assay Kits (Sinobestbio, Shanghai, China), respectively.

#### 2.5.4. Amino Acid Content

Mycelial powder (0.5 g) was extracted with 5 mL borate buffer (0.1 mol/L, pH 8.5) at 4 °C for 12–16 h, followed by sonication for 60 min. After centrifugation at 10,000 rpm for 10 min at 4 °C, 0.2 mL supernatant was derivatized with 0.6 mL AQC reagent and 1.2 mL borate buffer at room temperature in the dark for 10 min. The solution was filtered (0.22 μm) and analyzed by HPLC (UltiMate 3000, Thermo Fisher Scientific, Waltham, MA, USA) using a Welch Ultimate^®^ Amino Acid column (4.6 mm × 250 mm, 5 μm). Separation was performed at 40 °C with a flow rate of 0.8 mL/min, injection volume of 5 μL, and detection at 254 nm, using sodium acetate-triethylamine buffer (mobile phase A) and 80% acetonitrile (mobile phase B) in gradient elution. Amino acid standards (1 mg/mL stock solutions) were used for quantification.

### 2.6. LC-MS/MS Analysis

Freeze-dried powder (100 mg) was extracted with 1 mL pre-cooled methanol–water (4:1, *v*/*v*, 0.1% formic acid) by vortexing and ultrasonication for 30 min in an ice bath. After centrifugation at 12,000 rpm for 15 min at 4 °C, the supernatant was collected for LC-MS analysis (Orbitrap Exploris 240, Thermo Fisher Scientific, Waltham, MA, USA).

Chromatographic separation was performed on a Waters ACQUITY UPLC HSS T3 column (1.8 μm, 2.1 × 100 mm) at 40 °C. The mobile phase consisted of water with 0.1% formic acid (A) and acetonitrile with 0.1% formic acid (B), at a flow rate of 0.40 mL/min. The gradient elution program was: 0–5 min, 95% A to 35% A; 5–6 min, 35% A to 1% A; 6–7.5 min, hold at 1% A; 7.5–10 min, return to 95% A for equilibration. MS analysis was performed using electrospray ionization in both positive and negative modes. Full scan range was *m*/*z* 84–1250 at a resolution of 35,000. Key ion source parameters were: ion spray voltage (3.5 kV for positive, 3.2 kV for negative), sheath gas 30 arb, auxiliary gas 5 arb, ion transfer tube temperature 320 °C, and vaporizer temperature 300 °C. Data-dependent acquisition with stepped collision energy (30, 40, 50 V) was employed for MS/MS fragmentation. Six biological replicates were performed for each strain. Quality control (QC) samples were prepared by pooling aliquots of all samples, and data quality was controlled using a standard threshold of relative standard deviation (RSD) < 30% to ensure the repeatability and stability of the data.

### 2.7. Statistics and Data Analysis

All experiments were performed in triplicate except for the LC-MS/MS analysis. Observed differences were assessed for significance using SPSS 23.0, with results reported as mean ± standard deviation (SD). Principal component analysis (PCA) and partial least squares discriminant analysis (PLS-DA) were performed using R software (version 4.4.0) to visualize group separation and identify differential metabolites. Model validity was assessed through permutation tests. Differential metabolites between the CN-01 and JSH17 groups were screened based on VIP > 1.0, *p* < 0.05, and fold changes (FC) exceeding 2 or below 0.5. Hierarchical cluster analysis (HCA) was performed using the ComplexHeatmap R package and visualized as heatmaps.

## 3. Results and Discussion

### 3.1. ITS Sequence Analysis

**Figure 1:**
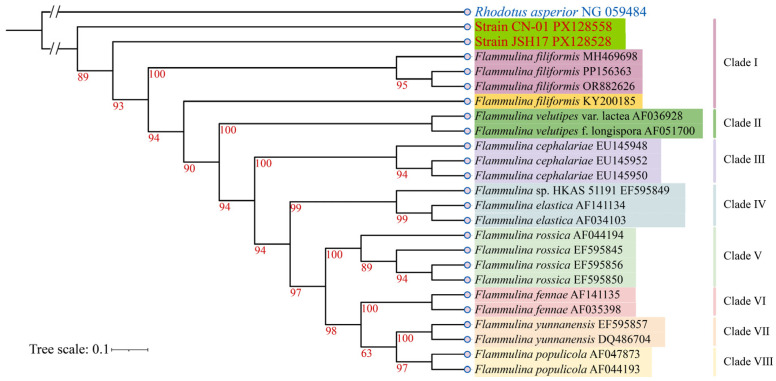
Bayesian phylogenetic tree based on ITS sequences.

Genomic DNA extracted from the fruiting bodies of strains CN-01 and JSH17 was used as a template for PCR amplification with universal primers ITS4 and ITS5. After sequencing verification, ITS sequences of 766 bp and 819 bp were obtained for CN-01 and JSH17, respectively. The assembled and corrected sequences were submitted to the GenBank database under accession numbers PX128558 (CN-01) and PX128528 (JSH17). Based on these ITS sequences, a phylogenetic tree was constructed using Bayesian inference (Figure 1). The analysis revealed that strains CN-01 and JSH17 clustered together on the same branch with high posterior probability, indicating a highly homologous genetic background and confirming that both belong to the species *Flammulina filiformis*.

### 3.2. Morphological Characteristics

**Figure 2:**
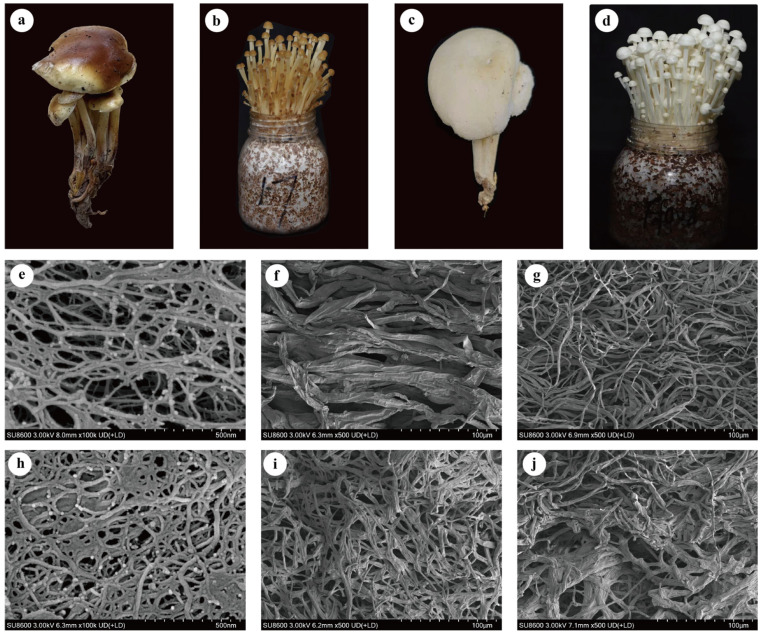
Morphological characteristics of wild-type *Flammulina filiformis* and their fruiting bodies. (**a**) Wild JSH17 strain; (**b**) JSH17 fruiting body; (**c**) wild CN-01 strain; (**d**) CN-01 fruiting body; (**e**) Ultrastructure of JSH17 pileus; (**f**) ultrastructure of JSH17 stipe; (**g**) ultrastructure of JSH17 stipe base; (**h**) ultrastructure of CN-01 pileus; (**i**) ultrastructure of CN-01 stipe; (**j**) ultrastructure of CN-01 stipe base.

The morphological features and microstructures of wild-type *F. filiformis* and their cultivated fruiting bodies are shown in Figure 2. Under natural conditions, the pileus of strain JSH17 exhibited brownish-yellow patches, with stipes in a naturally extended state (Figure 2a). In contrast, the pileus of strain CN-01 was white and relatively round, with stipes that were shorter and thicker (Figure 2b). However, after artificial cultivation and domestication, the morphology of the fruiting bodies differed significantly from their wild types. Strain JSH17 displayed slender stipes and a brownish pileus (Figure 2c), while strain CN-01 exhibited typical cultivated *F. filiformis* characteristics: slender stipes, densely clustered fruiting bodies, small pilei, and a pure white color (Figure 2d). Notably, under cultivation conditions, the entire growth cycle of strain JSH17 was 7 days shorter than that of strain CN-01.

Further ultrastructural observation of the cultivated fruiting bodies revealed distinct differences in microstructure between the two strains. As shown in Figure 2e–g, the pileus of strain JSH17 exhibited a loose porous network structure with relatively large and regularly arranged pores, representing a typical three-dimensional interconnected porous morphology [14]. Its stipe (sampled 1 cm from the pileus) was composed of thick, fibrous bundles, while the stipe base consisted of a network structure interwoven with finer fibers. In comparison, although the pileus of strain CN-01 also displayed a porous network, its structure was relatively denser with uneven pore size distribution (Figure 2h–j). Both the stipe and stipe base of CN-01 were composed of finer, more tightly arranged fibers, resulting in overall smaller pores. Therefore, the above morphological characteristics of the fruiting bodies (such as pileus and stipe features) further support the identification of both CN-01 and JSH17 as *F. filiformis*.

### 3.3. Electronic Tongue Analysis and Bioactive Compound Determination

**Figure 3:**
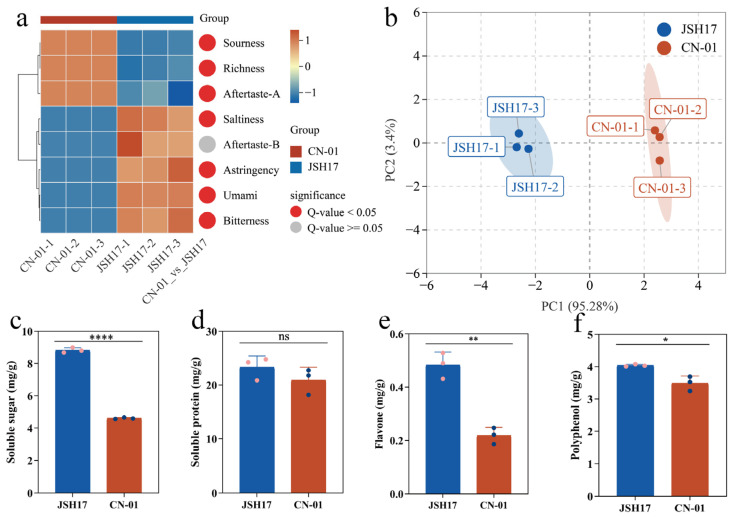
Electronic tongue analysis and bioactive components of *Flammulina filiformis*. (**a**) Electronic tongue analysis; (**b**) PCA of electronic tongue data; (**c**) soluble sugar content; (**d**) soluble protein content; (**e**) flavonoid content; (**f**) polyphenol content. Note: * indicates significant difference (ns: not statistically significant; *: *p* < 0.05; **: *p* < 0.01; ****: *p* < 0.0001).

To investigate differences in the main flavor profiles and bioactive components between the fruiting bodies of *F. filiformis* strains JSH17 and CN-01, electronic tongue analysis and determination of major active substances were performed. Electronic tongue results (Figure 3a) showed significant differences (*p* < 0.05) between JSH17 and CN-01 across multiple flavor dimensions, including Sourness, Richness, Aftertaste-A, Saltiness, Astringency, Umami, and Bitterness. Specifically, CN-01 exhibited higher intensities in Sourness, Richness, and Aftertaste-A, while JSH17 demonstrated significantly higher values in Saltiness, Aftertaste-B, Astringency, Umami, and Bitterness compared to CN-01. Aftertaste-A is primarily used to evaluate the astringency or other similar unpleasant mouthfeel remaining after swallowing [15], whereas Aftertaste-B typically reflects the intensity of bitterness or astringency remaining after swallowing, showing a high correlation with the variation trends of Astringency and Bitterness [16]. Principal component analysis based on electronic tongue data was conducted to assess similarity between the two strains [17]. The PCA results further indicated a clear separation in flavor composition between JSH17 and CN-01 (Figure 3b), with the first principal component (PC1) and second principal component (PC2) contributing 96.67% and 2.55% of the variance, respectively, confirming distinct overall flavor profiles.

Regarding bioactive components [18], the contents of soluble sugars, soluble protein, flavonoids, and polyphenol in strain JSH17 were 8.83, 23.30, 0.48, and 4.04 mg/g, respectively, while those in strain CN-01 were 4.60, 20.90, 0.22, and 3.49 mg/g, respectively (Figure 3c–f). Statistical analysis revealed that JSH17 had significantly higher soluble sugar, flavonoid, and polyphenol contents than CN-01, being 1.92-fold, 2.22-fold, and 1.16-fold higher, respectively. No significant difference was observed in soluble protein content between the two strains. Overall, JSH17 exhibited superior levels of multiple bioactive substances compared to CN-01, suggesting that it may possess richer bioactive components.

### 3.4. Amino Acid Content Determination

The content and composition of free amino acids (FAAs) in *F. filiformis* directly influence its flavor characteristics and nutritional value [19]. Systematic analysis of FAA contents in strains JSH17 and CN-01 (Table 1) revealed that the total FAA content of CN-01 (211.05 ± 16.37 mg/g) was significantly higher than that of JSH17 (191.75 ± 10.89 mg/g) (*p* < 0.05).

Different types of amino acids interact with taste receptors on the tongue due to their distinct molecular structures, thus eliciting specific tastes. A total of 18 amino acids were identified in both strains and classified according to their taste characteristics into umami (Asp, Glu), sweet (Thr, Ser, Gly, Ala, Pro, Asn, Gln), and bitter (Lys, Val, Met, Tyr, His, Arg, Cys, Trp, Phe) amino acids [20]. Umami amino acids (TUAA) are responsible for providing umami, contributing to savory, meaty, and mellow flavors. The key umami amino acid Glu exhibited the highest content in JSH17 (22.53 ± 1.15 mg/g, *p* < 0.01), and its TUAA content (26.90 ± 1.27 mg/g) was also significantly higher (*p* < 0.01), suggesting that JSH17 possesses greater umami potential. Sweet amino acids (TSAA) contribute to sweet taste, offering a mild and rounded mouthfeel. Among sweet amino acids, Gln and Ser—the most abundant—were significantly enriched in CN-01, with contents of 28.51 ± 3.23 mg/g and 14.59 ± 1.88 mg/g, respectively, compared to 22.06 ± 2.49 mg/g and 4.39 ± 0.13 mg/g in JSH17 (*p* < 0.01). Consequently, the TSAA content in CN-01 reached 77.26 ± 5.54 mg/g, 1.65-fold higher than that in JSH17 (46.79 ± 4.26 mg/g). Bitter amino acids (TBAA) are associated with bitter taste; although most are essential amino acids, they can produce unpleasant bitterness at high concentrations. For bitter amino acids, Val and Tyr were the most abundant in both strains, with contents of 53.06 ± 4.17 mg/g and 54.93 ± 8.33 mg/g in JSH17 and CN-01, respectively, corresponding to TBAA contents of 118.66 ± 7.25 mg/g and 100.62 ± 10.12 mg/g. Overall, the FAA composition of JSH17 was more oriented towards umami and bitter tastes, while CN-01 exhibited characteristics oriented towards umami and sweet tastes.

The specific flavor characteristics of food depend on both the content of flavor compounds and their threshold values [21]. To further evaluate the actual contribution of amino acids to the flavor of *F. filiformis*, the threshold-adjusted value (TAV), defined as the ratio of concentration to threshold, was calculated for each amino acid. Specifically, TAV > 1 indicates that the compound significantly contributes to the overall flavor, with its influence increasing as the TAV rises [22]. As shown in Table 1, the key umami amino acid Glu had TAVs greater than 1 in both strains, and the TAV in JSH17 (4.506 ± 0.230) was significantly higher than that in CN-01 (3.528 ± 0.046), further confirming its higher umami intensity (*p* < 0.01). Additionally, Val was the only amino acid with a TAV exceeding 1 exclusively in JSH17 (1.327 ± 0.104), indicating its contribution to perceptible bitterness. These findings are consistent with the amino acid composition analysis described above and corroborate the stronger signal responses observed for umami and bitterness dimensions in the electronic tongue measurement of JSH17.

### 3.5. Metabolomics Analysis

#### 3.5.1. Metabolite Composition Analysis

**Figure 4:**
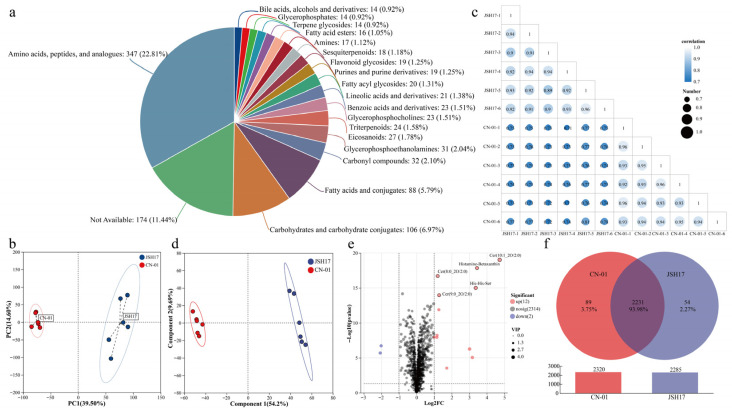
Composition analysis of metabolites in *Flammulina filiformis*. (**a**) Classification of metabolites; (**b**) PCA of metabolites; (**c**) correlation analysis between samples (with 6 biological replicates); (**d**) PLS-DA of metabolites; (**e**) volcano plot of differential metabolites; (**f**) Venn diagram analysis of metabolites.

To elucidate the differences in metabolite composition between the fruiting bodies of strains CN-01 and JSH17, a systematic comparison was performed using untargeted metabolomics technology. A total of 16,069 metabolic features were detected in positive and negative ion modes, and after database annotation and comparison, 2328 known metabolites were ultimately identified. Classification based on the HMDB database (Figure 4a) revealed a rich and diverse metabolite composition, comprising 22.8% amino acids, peptides, and analogues; 6.97% carbohydrates and carbohydrate conjugates; 5.79% fatty acids and conjugates; 2.1% carbonyl compounds; 2.04% glycerophosphoethanolamines; 1.78% eicosanoids; 1.58% triterpenoids; 1.51% glycerophosphocholines; 1.51% benzoic acids and derivatives; 1.38% lineolic acids and derivatives; 1.31% fatty acyl glycosides; 1.25% purines and purine derivatives; 1.25% flavonoid glycosides; 1.18% sesquiterpenoids; 1.12% amines; 1.05% fatty acid esters; 0.92% terpene glycosides; 0.92% glycerophosphates; and 0.92% bile acids, alcohols and derivatives. Among these, amino acids, peptides, and analogues constituted the largest group with 347 compounds, followed by carbohydrates and carbohydrate conjugates as the second largest group with 106 compounds identified. Amino acids and analogues are key regulators of plant development and critical factors in preventing and resisting biotic and abiotic stresses [23]. Furthermore, the flavor and aroma of edible fungi are inseparable from umami amino acids and aromatic compounds [24].

PCA was performed on the CN-01 and JSH17 groups. All samples were located within the 95% confidence interval, with PC1 and PC2 contributing 37.10% and 13.30% of the variance, respectively, for a cumulative contribution of 49.40% (Figure 4b). The PCA score plot clearly showed good clustering within each group (CN-01 and JSH17) and distinct separation between groups, indicating significant differences in the metabolic profiles of the two strains. Correlation analysis between samples further supported this conclusion, with inter-group correlation coefficients generally below 0.9 (Figure 4c). To enhance discrimination between groups and screen for differential metabolites, PLS-DA was subsequently performed. As shown in Figure 4d, the model exhibited good explanatory and predictive power (R^2^X = 0.673, R^2^Y = 0.992, Q^2^ = 0.966), with Q^2^ > 0.9 indicating excellent predictive reliability [25] and suitability for differential metabolite analysis.

Differential metabolite screening was conducted using a combination of univariate analysis and PLS-DA, with results visualized using volcano plots and Venn diagrams. Volcano plots were used to display differences in the relative abundance of metabolites between the two comparison groups [26]. Based on the criteria of *p* < 0.05, VIP > 1.0, and FC exceeding 2 or below 0.5, a total of 14 differential metabolites were identified between the CN-01 and JSH17 groups, of which 12 were relatively upregulated, and 2 were downregulated in CN-01 compared to JSH17 (Figure 4e). Compounds such as amino acids, peptides, and analogues, as well as fatty acids and conjugates, predominated among these differential metabolites. A Venn diagram was used to examine the interrelationships among metabolites detected in each group. As shown in Figure 4f, 2231 metabolites were shared between the CN-01 and JSH17 groups, accounting for 93.98% of the total known metabolites. The number of unique metabolites in the CN-01 and JSH17 groups was 89 (3.75%) and 54 (2.27%), respectively, and the total number of identified features in the CN-01 group (2320) was slightly higher than that in the JSH17 group (2285). These results indicate that the overall metabolite composition of the fruiting bodies of the two strains is highly similar; however, a certain number of differential metabolites still exist, which may be associated with their phenotypic differentiation.

#### 3.5.2. Metabolite Clustering Analysis

**Figure 5:**
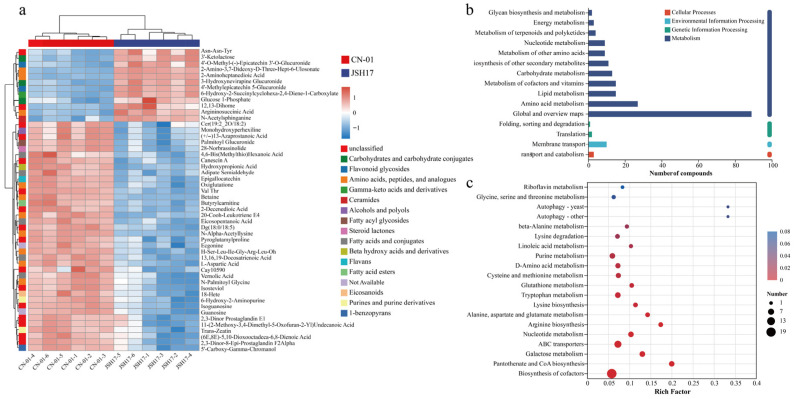
Cluster analysis and KEGG pathway analysis of metabolites. (**a**) Cluster heatmap analysis of metabolites (red indicates upregulation, blue indicates downregulation); (**b**) KEGG second-level pathway analysis; (**c**) KEGG pathway enrichment analysis.

To further elucidate the metabolic differences between the fruiting bodies of strains CN-01 and JSH17, hierarchical clustering analysis was performed on the top 50 most significantly differential metabolites between the two groups [27] (Figure 5a). The results revealed a clear divergence in expression patterns of these metabolites between the CN-01 and JSH17 groups.

The metabolites significantly enriched in the CN-01 group mainly included eight amino acids, peptides, and analogues (e.g., Val-Thr, N-Palmitoyl Glycine, L-Aspartic Acid, H-Ser-Leu-Ile-Gly-Arg-Leu), five fatty acids and conjugates (Eicosopentanoic Acid, Adipate Semialdehyde, 13,16,19-Docosatrienoic Acid, Vernolic Acid), one steroid lactone (28-Norbrassinolide), one flavan (Epigallocatechin), two purines and purine derivatives (6-Hydroxy-2-Aminopurine and Trans-Zeatin), and one 1-benzopyran (5′-Carboxy-Gamma-Chromanol). Most of these substances are derivatives of amino acids and fatty acids. The large quantities of amino acids and lipids accumulated during the growth phase of fungi such as *F. filiformis* serve as important structural substrates and energy reserves, driving subsequent development and differentiation. The rapid growth of *F. filiformis* mycelia is highly dependent on short-chain peptides (e.g., leucine and glycine) and free amino acids (e.g., isoleucine and threonine), which function not only as raw materials for protein synthesis but also as key signaling molecules [28]. 28-Norbrassinolide, a brassinosteroid analogue, is recognized as the sixth major plant hormone and possesses multiple physiological functions, including promoting growth and enhancing stress resistance [29]. Epigallocatechin, a naturally occurring polyphenol, exhibits antioxidant, antitumor, and antiviral activities, and plays a role in preventing diabetes [30]. Zeatin is a plant growth regulator existing in both cis and trans isomeric forms [31], with Trans-Zeatin exhibiting significantly higher biological activity than Cis-Zeatin.

In contrast, the metabolites significantly enriched in the JSH17 group mainly included four amino acids, peptides, and analogues (e.g., Asn-Asn-Tyr, Argininosuccinic Acid, 2-Aminoheptanedioic Acid), three carbohydrates and carbohydrate conjugates (Glucose 1-Phosphate, 3′-Ketolactose, 3-Hydroxynevirapine Glucuronide), two flavonoid glycosides (4′-O-Methyl-(-)-Epicatechin 3′-O-Glucuronide, 4′-Methylepicatechin 5-Glucuronide), one ceramide (N-Acetylsphinganine), and one unclassified compound (12,13-Dihome). Among these, Glucose 1-Phosphate (G1P), generated from glycogen breakdown by glycogen phosphorylase or converted from glucose-6-phosphate by phosphoglucomutase, is a key intermediate in glycogen metabolism and carbohydrate biosynthesis [32]. Flavonoids are a class of compounds widely present in *F. filiformis*, mostly existing as glycosides conjugated with sugars. Flavonoid glycosides, formed by the conjugation of flavonoids with sugars such as glucose and rhamnose, are natural pigments possessing various biological activities [33]. N-Acetylsphinganine, a ceramide, can modulate protein phosphatase activity and is involved in cell differentiation and apoptosis processes. 12,13-Dihome, a small molecule derived from linoleic acid metabolism, plays an important role in energy metabolism [34]. In summary, the clustering analysis revealed distinct metabolic fluxes and accumulation characteristics between the two strains in amino acid/peptide metabolism, lipid metabolism, and secondary product synthesis pathways, providing a basis for explaining their differences in physiological functions.

#### 3.5.3. KEGG Pathway Analysis

To further investigate the differences in metabolic pathways between the fruiting bodies of *F. filiformis* strains CN-01 and JSH17, the identified differential metabolites were mapped to the KEGG database for pathway enrichment analysis. As shown in Figure 5b, the differential metabolites were significantly enriched in 15 s-level pathways, primarily belonging to Metabolism, followed by Genetic Information Processing, Environmental Information Processing, and Cellular Processes. In-depth analysis of the top 20 pathways ranked by enrichment significance (Figure 5c) revealed that these pathways encompassed Amino acid metabolism (9 pathways), Metabolism of cofactors and vitamins (2 pathways), Nucleotide metabolism (2 pathways), as well as one pathway each for Carbohydrate metabolism and Lipid metabolism. Among these, the metabolic pathways exhibiting particularly significant differences (*p* < 0.01) between the CN-01 and JSH17 groups included: Biosynthesis of cofactors, Pantothenate and CoA biosynthesis, Galactose metabolism, ABC transporters, Nucleotide metabolism, Arginine biosynthesis, Alanine, aspartate and glutamate metabolism, Lysine biosynthesis, Tryptophan metabolism, Glutathione metabolism, Cysteine and methionine metabolism, and D-Amino acid metabolism.

These enriched pathways reveal key metabolic characteristics during fruiting body development in the two strains. The enrichment of the Pantothenate and CoA biosynthesis pathway indicates enhanced coenzyme A synthesis, which is associated with fatty acid metabolism and energy production [35]. Upregulation of carbohydrate metabolism (e.g., Galactose metabolism) and nucleotide metabolism collectively reflects the high demand for ATP and structural precursors during the rapid growth phase of fruiting bodies [36]. The significant enrichment of various amino acid metabolism pathways (e.g., Arginine biosynthesis, Alanine, aspartate and glutamate metabolism) is closely related to protein and enzyme synthesis [37], as well as the accumulation of flavor compounds during fruiting body development [38]. Notably, enhancement of the Glutathione metabolism pathway may contribute to improved antioxidant capacity, helping the organism cope with reactive oxygen species accumulation [39]. Furthermore, according to literature reports [4], the biosynthesis pathways of phenylalanine, tyrosine, and tryptophan are associated with pigment formation in *F. filiformis*. However, in the present study, these pathways were not found to be significantly enriched in the white strain CN-01, which may be a crucial metabolic basis for the maintenance of its white fruiting body phenotype. In summary, KEGG pathway analysis indicates systematic differences between the two *F. filiformis* strains in energy metabolism, amino acid conversion, and antioxidant balance, and these differences may collectively influence their growth characteristics, appearance color, and flavor quality.

#### 3.5.4. Metabolic Pathway Analysis

**Figure 6:**
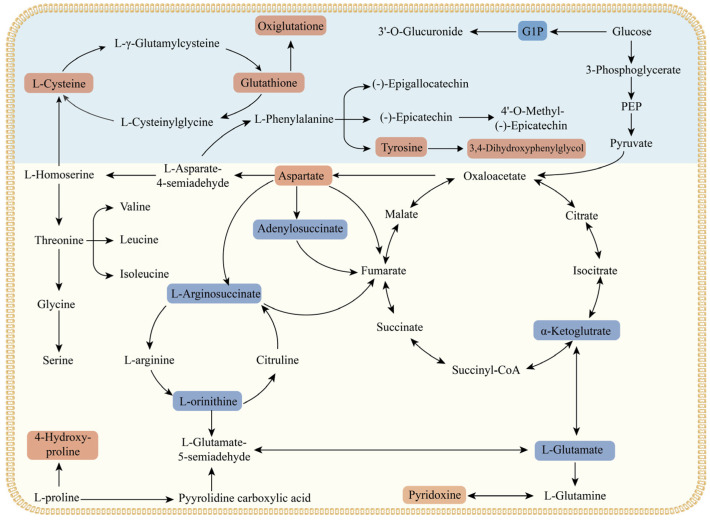
Comprehensive metabolic regulatory pathways in *Flammulina filiformis* analyzed by metabolomics. Raw metabolomics data are displayed with metabolite names. Red squares indicate upregulation, and blue squares indicate downregulation.

To elucidate the differences in metabolic pathways between *F. filiformis* fruiting bodies of strains CN-01 and JSH17, the significantly enriched pathways (*p* < 0.05) were mapped and visually analyzed [40]. As shown in Figure 6, differential metabolites were primarily enriched in pathways related to amino acid metabolism, energy metabolism, and secondary metabolite biosynthesis. The alanine, aspartate, and glutamate metabolism pathway was significantly enriched between the two strains, wherein the key umami amino acid L-Aspartic Acid was significantly upregulated in the white strain CN-01, while intermediates such as Argininosuccinic Acid, L-Ornithine, and 2-Oxoglutaric Acid were downregulated, indicating divergent metabolic flux within this pathway between the two strains. Enrichment of the glutathione metabolism pathway suggested that the yellow strain JSH17 might face stronger oxidative stress during fruiting body development, enhancing its antioxidant defense capacity through upregulation of glutathione-related metabolites [39], which is consistent with its shorter growth cycle and higher metabolic activity.

Energy metabolism-related pathways also exhibited significant differences. Enrichment of the pantothenate and CoA biosynthesis pathway indicated differences in energy metabolism demands between the two strains. Coenzyme A, as a core cofactor in the tricarboxylic acid cycle and fatty acid metabolism, has its biosynthesis pathway activity directly linked to energy supply efficiency [35]. Compared to JSH17, differential metabolites in CN-01 were not significantly enriched in the aromatic amino acid biosynthesis pathways (phenylalanine, tyrosine, and tryptophan metabolism), which serve as starting points for the synthesis of pigment compounds such as flavonoids and melanins [41]. In summary, the white strain CN-01 tended to allocate metabolic resources towards amino acid accumulation and umami substance synthesis, while the yellow strain JSH17 exhibited stronger energy metabolism and antioxidant metabolic activity, with the activation of pigment synthesis-related pathways forming the metabolic basis for its yellow phenotype formation. The systematic differences between the two strains in primary and secondary metabolic networks collectively constitute the underlying biochemical mechanisms for their color differentiation and quality variation.

## 4. Discussion

*Flammulina filiformis* is an important cultivated edible mushroom in China, primarily comprising yellow and white strains. Among them, the artificially bred “albino” strains originated from Japan [42], while the yellow strains were domesticated from wild isolates. Currently, crossing and selective breeding using yellow and white strains as parents have yielded some high-yield, high-quality light-yellow varieties. However, reports on naturally occurring white *F. filiformis* strains native to China remain scarce.

White and yellow *F. filiformis* differ significantly in yield, quality, and texture, leading to differences in their commercial value [5]. In this study, we found that the CN-01 strain exhibited significantly higher total FAA content than JSH17, while its total phenolic and total flavonoid contents were lower. This seemingly contradictory pattern actually reflects differential resource allocation within the metabolic networks of the two strains, revealing distinct metabolic strategies and functional orientations. CN-01 showed greater activity in amino acid metabolism pathways (e.g., alanine, aspartate, and glutamate metabolism), suggesting that its metabolic flux is preferentially directed toward protein synthesis and flavor compound accumulation. In contrast, JSH17 exhibited significant enrichment in pathways such as glutathione metabolism and aromatic amino acid biosynthesis, which are closely associated with the synthesis of secondary metabolites, including flavonoids, phenolics, and pigments. This differential allocation of metabolic flux may reflect distinct survival strategies shaped during evolution or domestication. CN-01 appears to allocate resources preferentially toward primary metabolism directly related to flavor, whereas JSH17 has enhanced secondary metabolism linked to antioxidant defense and stress responses. Future research could combine molecular marker-assisted breeding and cross-breeding to promote the accumulation of bioactive metabolites while maintaining desirable color and flavor traits, thereby developing novel *F. filiformis* varieties with high nutritional value and excellent commercial characteristics.

Metabolomics, which directly captures phenotypic traits, has become a widely used approach in studies on color variation within the same species. Lv et al. [23] employed metabolomics to profile yellow and white fruiting bodies of *F. filiformis* and identified flavonoids as key pigment components. Zhou et al. [43] found that color differences in different eggplant varieties were associated with significant enrichment in the phenylpropanoid biosynthesis and arginine biosynthesis pathways. Yusoff et al. [44] analyzed the chemical and physical properties of extracts from *Flammulina filiformis* and other species; high-performance liquid chromatography (HPLC) analysis suggested that these extracts may contain eumelanin and pheomelanin. Im et al. [4], by cloning and comparing the gene sequences encoding phenylalanine ammonia-lyase in different colored *F. filiformis* strains, proposed that this gene serves as a key determinant of color variation. In a similar vein, our study found that the CN-01 strain did not exhibit significant enrichment in the metabolic pathways of phenylalanine, tyrosine, and tryptophan, which may represent the metabolic basis for insufficient accumulation of pigment precursors, ultimately resulting in the white fruiting body phenotype. However, the activity and expression levels of pigment synthases downstream of the aromatic amino acid synthesis pathway—such as tyrosinase, polyphenol oxidase [45], and chalcone synthase [46]—also directly influence the final pigment output. Therefore, future integration of multi-omics approaches, including pan-genomics, transcriptomics, and genome-wide association studies (GWASs), will enable more precise elucidation of the mechanisms underlying trait variation.

In this study, we integrated molecular identification, electronic tongue analysis, determination of amino acids and bioactive components, and untargeted metabolomics to systematically compare the naturally occurring white variant CN-01 with the yellow strain JSH17. Molecular identification and morphological observation confirmed that both strains belong to *Flammulina filiformis*. Nutritional quality analysis revealed that the FAA content of CN-01 was significantly higher than that of JSH17, with a flavor profile dominated by umami and sweet tastes. Metabolomics analysis further indicated that while the overall metabolite composition of the two strains was highly similar, the significantly differential metabolites were primarily enriched in pathways related to amino acid metabolism (e.g., alanine, aspartate, and glutamate metabolism), energy metabolism (e.g., pantothenate and CoA biosynthesis), and glutathione metabolism. This study provides new targets and germplasm resources for color breeding and quality improvement of edible fungi, and holds important theoretical value and application prospects for promoting germplasm innovation of white *F. filiformis* in China. Furthermore, the naturally occurring white variant strain exhibits potential application value in low-sodium condiments, flavor enhancers, and functional foods targeting specific populations (e.g., hypertensive patients and the elderly).

## Figures and Tables

**Table 1:** Types and contents of FAAs in *Flammulina filiformis*.

Taste	Free Amino Acid	Flavor Threshold (mg/g)	Free Amino Acid Content (mg/g)		TAV	
			JSH17	CN-01	JSH17	CN-01
Umami	Asp	100	4.37 ± 0.25	5.52 ± 0.36 **	0.044	0.055 *
	Glu	5	22.53 ± 1.15	17.64 ± 0.23 **	4.506	3.528 **
	**TUAA**		26.90 ± 1.27	23.16 ± 0.43 **		
Sweet	Thr	260	3.95 ± 0.28	4.46 ± 0.51 *	0.015	0.017 *
	Ser	150	4.39 ± 0.13	14.59 ± 1.88 **	0.029	0.097 **
	Gly	130	2.12 ± 0.50	7.80 ± 0.89 **	0.016	0.060 **
	Ala	60	5.75 ± 3.01	7.02 ± 0.32 *	0.096	0.117 *
	Pro	300	2.94 ± 0.32	7.38 ± 1.05 **	0.010	0.025 **
	Asn	100	5.58 ± 0.53	7.50 ± 0.91 **	0.056	0.075 **
	Gln	250	22.06 ± 2.49	28.51 ± 3.23 **	0.088	0.114 **
	**TSAA**		46.79 ± 4.26	77.26 ± 5.54 **		
Bitter	Lys	50	8.48 ± 1.09	9.77 ± 1.39 *	0.170	0.195 *
	Val	40	53.06 ± 4.17	6.36 ± 0.87 **	1.327	0.159 **
	Met	30	5.47 ± 0.69	2.37 ± 0.05 **	0.182	0.079 **
	Tyr	91	13.77 ± 1.37	54.93 ± 8.33 **	0.151	0.604 **
	His	20	2.67 ± 0.30	3.87 ± 0.43 **	0.134	0.194 **
	Arg	50	10.33 ± 2.46	9.63 ± 1.47	0.207	0.193
	Cys	-	9.96 ± 1.02	15.06 ± 1.92 **	-	-
	Trp	90	9.68 ± 1.47	6.81 ± 0.78 *	0.108	0.076 *
	Phe	90	4.64 ± 0.38	1.83 ± 0.05 **	0.052	0.020 **
	**TBAA**		118.66 ± 7.25	100.62 ± 10.12 *		
	**FAAs**		191.75 ± 10.89	211.05 ± 16.37 *		

Note: * indicates significant difference (*: *p* < 0.05; **: *p* < 0.01); TAV = amino acid/taste threshold; TUAA: total umami amino acid; TSAA: total sweet amino acid; TBAA: total bitter amino acid; FAAs: total free amino acids. Threshold is the lowest taste concentration of amino acids; -: no threshold.

## Data Availability

The original contributions presented in this study are included in the article. Further inquiries can be directed to the corresponding authors.
